# Metacognitive reflections on trajectory strategies in mixed reality: real-time and retrospective reports during mental jigsaw puzzles

**DOI:** 10.1007/s00426-026-02286-6

**Published:** 2026-04-09

**Authors:** Tsuyoshi Yoshioka, Jun Saiki

**Affiliations:** Graduate School of Human and Environmental Studies, Kyoto University, Yoshida-nihonmatsu-cho, Sakyo-ku, Kyoto, 606-8501 Japan

**Keywords:** Mental translation, Mental rotation, Jigsaw puzzles, Metacognition, Metacognitive strategies, Embodied cognition

## Abstract

Real-world object fitting often requires indirect, detour-like trajectories due to physical constraints, such as lifting and maneuvering physical puzzle pieces. Reflecting this, recent work using three-dimensional puzzle stimuli has reported asymmetric reaction time (RT) patterns around 180° in fitting tasks—and unexpectedly, even in classical mental rotation tasks that require object matching. This finding challenges the classical assumption that equal angular disparities yield equivalent RTs. One possible interpretation is that RTs reflect not only rotational but also translational components of mental manipulations; however, classical matching tasks contain no physical obstacles that would necessitate detour-like trajectories. Thus, behavioral RT data alone are insufficient for determining whether detour-like mental translations are actually involved. To address this limitation, the present study incorporated metacognitive reflection on translational trajectories during both fitting tasks and classical matching tasks. Participants first completed both tasks under speed–accuracy instructions, after which they retrospectively selected the animation (shortcut vs. detour trajectories) that best represented their strategies during these earlier cognitive tasks. These retrospective reflection reports were compared with real-time reflection reports to assess their malleability. Replicating previous work, RTs showed robust asymmetries around 180° in both tasks. Crucially, subjective strategy reports diverged from these behavioral patterns: retrospective reflections mirrored RT asymmetries in the fitting task but not in the classical matching task. Moreover, metacognitive reflections were malleable in the fitting task, whereas they remained stable in the matching task.

## Introduction

Metacognition—the ability to monitor and control one’s own cognitive processes—plays a foundational role in effective thinking and problem solving (Flavell, 1979). According to Nelson and Narens’s (1990) framework, metacognition consists of two interrelated components: monitoring (assessing the current state of cognition) and control (adjusting behavior based on that assessment). Schraw and Moshman (1995) further distinguished between metacognitive knowledge—including declarative, procedural, and conditional components—and metacognitive regulation, which involves planning, monitoring, and evaluating cognitive activity. Importantly, they also proposed a developmental framework comprising tacit, informal, and formal metacognitive theories, reflecting increasing levels of awareness, reflection, and theorization in how individuals understand their own cognition (Schraw & Moshman, 1995).

The practical relevance of metacognition is well documented in educational contexts, where metacognitive learning strategies such as practice testing that involves self-testing, spaced practice, and interleaved practice are linked to improved learning outcomes (Bjork et al., 2013; Dunlosky et al., 2013; Stanton et al., 2021). Among these, distributing learning over time has been rated as a highly effective strategy for retention (Dunlosky et al., 2013). However, metacognitive processes also exhibit well-documented limitations, particularly the tendency toward overconfidence. In a classical study, Fischhoff et al. (1977) found that university students often endorsed incorrect general-knowledge answers with strong confidence.

Because metacognitive processes are not directly observable, researchers have relied on a range of indirect methods to infer how individuals monitor and regulate their thinking. Self-report questionnaires offer introspective access to cognitive processes such as perceived strategy use and metacognitive awareness (Schraw & Dennison, 1994), but are susceptible to memory distortions and response biases. Think-aloud protocols provide real-time verbal data, though the act of verbalization—such as explaining reasons—may itself influence performance (Ericsson & Simon, 1993). Observation-based assessments—where trained evaluators code behavioral indicators of metacognition—have been used to circumvent limitations in the literacy demands of self-report measures (Gascoine et al., 2017). Nevertheless, each of these methods captures metacognition only indirectly. Therefore, interpretations should be made with caution, as subjective findings may not always align with objective performance (e.g., overconfidence).

However, overconfidence is not uniform across cognitive domains, and its expression may vary depending on the type of task. One such domain is mental rotation—an important component of spatial cognition—that has received far less attention from a metacognitive perspective. Shepard and Metzler’s (1971) classical mental rotation task has inspired decades of research into mental manipulations, yet relatively few studies have examined whether individuals can accurately reflect on the strategies they use during such tasks. Among the limited work on metacognition, some studies have used confidence as an index of metacognitive monitoring, with findings showing moderate to strong confidence–performance correlations in variants of mental rotation tasks (Cooke-Simpson & Voyer, 2007; Estes & Felker, 2012).

More recent work has expanded beyond mental rotation to examine metacognitive monitoring across a broader range of spatial domains, offering a more general perspective on how individuals evaluate their performance. For example, Ariel and Moffat (2018) examined age-related differences in confidence across spatial tasks such as mental rotation and paper folding. They found that older adults showed lower performance and lower confidence than younger adults, although their monitoring accuracy (i.e., the alignment between subjective confidence and objective accuracy) was mostly preserved (Ariel & Moffat, 2018). In another study, Ariel et al. (2018) also examined gender differences among university students across spatial tasks and found that females often expressed lower confidence than males. Notably, this confidence gap emerged even in a task where performance did not differ significantly between their genders (i.e., the paper-folding test). At the same time, their results indicated that monitoring accuracy showed minimal gender differences across most tasks (Ariel et al., 2018), contrasting with earlier mental rotation research reporting gender differences in monitoring accuracy (Cooke-Simpson & Voyer, 2007).

Taken together, this body of literature highlights a key gap. Specifically, most metacognitive studies in spatial cognition focus on end-state confidence judgments—that is, how confident participants are in their final answers—rather than on how individuals reflect on the intermediate mental operations that support spatial reasoning, such as imagined movement paths.

Importantly, confidence and performance findings in prior spatial cognition research (Ariel et al., 2018; Ariel & Moffat, 2018; Cooke-Simpson & Voyer, 2007) underscore the importance of task structure in shaping spatial processing, thereby drawing renewed attention to mental rotation tasks. Unlike spatial tasks such as paper folding, which closely reflect physically realizable actions governed by physical constraints (e.g., material properties, occlusion), the classical mental rotation task introduced by Shepard and Metzler (1971) requires participants to make same–different matching judgments about two objects. As a model of real-world object manipulation—particularly fitting-like actions that are shaped by physically relevant constraints (e.g., obstructions, insertion difficulty)—this format has limited ecological validity because it does not incorporate such constraints. Addressing these limitations requires novel task designs that employ more realistic spatial scenarios and constraints.

To address the limitations of conventional mental rotation paradigms and better reflect real-world object interactions, recent research has introduced mental jigsaw puzzles as a more ecologically grounded alternative (Yoshioka, 2023; Yoshioka et al., 2025). These tasks provide a framework for investigating how spatial strategies emerge in contexts involving the integration of multiple components, rather than mere shape matching or rotation. Contrasting with classical mental rotation tasks, which use a pair of T-type objects and require object matching, mental jigsaw puzzles use a pair of U-type and T-type objects and require object fitting, a difference that may elicit distinct strategies (Yoshioka et al., 2025). Indeed, eye-tracking and self-report data suggest that participants tend to mentally reposition smaller pieces toward larger, more stable assemblies, exhibiting directional biases (Yoshioka et al., 2025).

These distinct directional strategies help distinguish between matching (alignment) in mental rotation tasks and fitting (insertion) in mental jigsaw puzzles (Yoshioka, 2023; Yoshioka et al., 2025)—terms often treated interchangeably in prior literature (Frick et al., 2013; Frick & Pichelmann, 2023; Mutlu et al., 2020). For example, in their jigsaw puzzle tasks, Mutlu et al. (2020) instructed participants to fit a piece into the central space of a puzzle assembly, yet labeled the condition as a “match” task. Similarly, Frick et al. (2013) and Frick and Pichelmann (2023) used language implying insertion (“fitting pieces into holes”) while referring to the process as matching.

These jigsaw puzzle studies (Frick et al., 2013; Frick & Pichelmann, 2023; Mutlu et al., 2020) have primarily focused on the start and end points of imagined movements, paying limited attention to how individuals mentally handle intermediate trajectories—especially under spatial constraints. To investigate this middle stage, Yoshioka et al. (2025) examined translational trajectories by analyzing reaction time (RT) patterns, contrasting shortcut trajectories (straight-line translation paths regardless of implied obstacles) with detour trajectories (curve-line translation paths that avoid implied obstacles). Although mental rotation and mental jigsaw puzzle tasks share identical start and end points, they differ in whether physical constraints are implied during mental manipulations.

Contrary to predictions, Yoshioka et al. (2025) found that both tasks exhibited asymmetric RT patterns around 180°, with longer RTs at angles implying greater physical constraint (e.g., 300°) than at angles with fewer constraints (e.g., 60°). While this asymmetry is consistent with detour-like trajectories in mental jigsaw puzzles, its presence in classical mental rotation tasks was unexpected and difficult to reconcile with purely rotation-based accounts. One possible interpretation is to extend the classical account proposed by Shepard and Metzler (1971) by assuming that, in addition to rotational distance, translational distance may also contribute to RTs, thereby producing detour-like patterns even in mental rotation tasks (Yoshioka et al., 2025). However, this interpretation lacks a clear rationale, particularly because no physical obstacles are present in the classical mental rotation task that would necessitate detour-like trajectories. As a result, RT data alone proved insufficient for determining whether participants actually engaged in detour-like mental translations.

This ambiguity highlights the need for replication, particularly in light of broader concerns about replicability in psychological science (Open Science Collaboration, 2015), as the observed asymmetry directly questions a core assumption in mental rotation research—namely, angular-disparity equivalence. At the same time, it underscores the need for complementary approaches that directly access how individuals conceptualize their spatial manipulation strategies. Accordingly, the present study replicates the RT findings of Yoshioka et al. (2025) using a mixed reality (MR) environment with more ecologically grounded stereoscopic depth cues, while extending this approach by incorporating metacognitive reflection to directly probe participants’ subjective representations of translational trajectories.

Within this replication framework, we introduce metacognitive reflection—a less explored area in mental rotation research—as a way to access how individuals conceptualize their mental strategies during these tasks. Rather than inferring strategy from behavior alone, we ask participants to explicitly report which of two animated trajectories best represents their mental process (shortcut vs. detour paths). This approach bridges the gap between behavior and metacognitive monitoring, emphasizing subjective insight. Specifically, we explore whether participants reflect on detour paths when object configurations imply spatial obstacles, and whether these preferences differ between matching (alignment without obstacles) and fitting (insertion involving obstacles).

We focused on two central research questions. The first concerns what type of strategy participants reflect on when reasoning about object movement under physical constraints. One possibility is that participants reflect on strategies that simulate realistic object movement constrained by the physical structure of the environment, particularly U-type object in the fitting task. This view, referred to here as the physical-simulation hypothesis, suggests that participants reflect on mentally simulating how an object would move while accounting for implied obstacles, leading them to report more detour-like paths in fitting objects, where spatial constraints are prominent. This view aligns with affordance theory (Gibson, 1979), in which the environment provides action possibilities.

Alternatively, participants may reflect on a more abstract, geometry-based strategy that emphasizes internal alignment and visual projection over physical realism. According to this minimum-distance hypothesis, spatial reasoning is based on the principle of minimizing the distance between objects, regardless of physical obstacles. This is especially plausible in tasks involving object fitting or matching, which inherently rely on the spatial superimposition of shapes. In such cases, translation along the shortest path may be perceived as the most efficient and intuitive strategy. This perspective is consistent with previous research on jigsaw puzzle tasks, where the processes of fitting and matching are often treated as functionally interchangeable (Frick et al., 2013; Frick & Pichelmann, 2023; Mutlu et al., 2020). It also aligns with findings from imagery research, such as mental scanning (Kosslyn et al., 1978) and cognitive “tunneling” (Burke, 1952).

The second research question addresses the embodiment of strategy use—that is, whether metacognitive reflections are grounded in physical or cognitive forms of embodiment. Previous research has demonstrated that prior physical interactions can influence subsequent performance in mental rotation tasks. For instance, participants who had handled heavier objects beforehand exhibited slower response times in motor imagery tasks, while performance on visual imagery tasks remained unaffected (Flusberg & Boroditsky, 2011). These findings support the framework of embodied cognition, which posits that cognitive processes are deeply rooted in the body itself or in the body’s interactions with the environment (Shapiro & Spaulding, 2021). The notion of embodiment derives from this theoretical view.

Applied to mental jigsaw puzzles, fitting behaviors are highly familiar from everyday activities—such as inserting a puzzle piece into an assembly, placing items into a container, or navigating a car into a tight parking space. These everyday actions inherently involve avoiding obstacles and negotiating physical constraints. Because such experiences are repeated and sensorimotor in nature, it is plausible that people’s mental representations of “fitting” are already embodied. As a result, individuals may draw on these embodied regularities when introspecting about object movement, influencing the strategies they report in tasks that imply physical constraints. We refer to this as a physical embodiment hypothesis in the present study. Under this perspective, reflection patterns—such as a preference for detour trajectories—should remain stable even when more efficient shortcut trajectories are plausible, because they are grounded in internalized simulations of object movement.

In contrast, a cognitive embodiment hypothesis allows for a reinterpretation that does not solely rely on physically realistic motion. From this perspective, individuals may gravitate toward shortcut trajectories when they conceptualize the task at a more abstract level. Because the task ultimately requires only the spatial alignment of the start and end points of two objects, the entire translational path can be denoted by a single vector connecting these two points (e.g., point A → B). A shortcut trajectory therefore requires only this minimal geometric specification. By contrast, a detour trajectory—being curved—cannot be fully specified by two points alone and instead requires at least one additional intermediate point (e.g., point A → C → B), thereby increasing computational complexity. Thus, although detour paths are physically plausible, they may impose greater cognitive costs.

Taken together, the present study primarily investigates metacognitive reflection in mental jigsaw puzzle and mental rotation tasks, while also examining RT patterns to assess whether previously reported behavioral asymmetries are replicated. Specifically, the study addresses two core research questions concerning metacognitive reflection: **(1)** which type of translational trajectory participants retrospectively report (shortcut vs. detour; minimum-distance vs. physical-simulation hypotheses), and **(2)** whether these reflections are malleable across interpretive conditions (real-time vs. retrospective reflections; cognitive vs. physical embodiment hypotheses). Complementarily, the study explores the relationship between RT patterns and subjective strategy reports to examine their alignment in mental translation, motivated by classical findings showing that participants’ introspective reports were consistent with behavioral evidence interpreted as reflecting mental rotation processes (Shepard & Metzler, 1971).

### Detailed explanations on predictions

To empirically test the hypotheses, we derived the following predictions. To address the first question, we examine angular pair differences in retrospective reflection. If participants conceptualize mental jigsaw puzzles as involving purely geometric, physics-free mental manipulation, they are expected to treat obstacles as irrelevant. Under this minimum distance hypothesis, retrospective reflections should exhibit symmetry between mirror-symmetric rotation pairs around 180° (i.e., 60° vs. 300°, 120° vs. 240°), suggesting that strategy use is independent of physical constraints. Such symmetry around 180° is also expected in mental rotation tasks, where the absence of implied physical barriers may lead participants to rely on more abstract and geometric strategies. In contrast, if mental jigsaw puzzles elicit retrospective reflections grounded in physical operations, participants should report more detour trajectories that take implied obstacles into account, resulting in asymmetries across mirror-symmetric angles, with more detour reflections at 300° than 60°, and at 240° than 120°.

We also examine task differences in retrospective reflections. Specifically, we compare mental jigsaw puzzles and mental rotation tasks to determine whether participants retrospectively reflect on their strategy use in a manner consistent with geometrically efficient reasoning or with physically grounded, jigsaw-like problem solving. Under the physical simulation hypothesis, participants should retrospectively reflect on detour strategies more frequently in mental jigsaw puzzles than in mental rotation tasks. However, if both tasks are construed similarly—as assumed in the minimum distance hypothesis—no significant task-based differences in retrospective reflections are expected.

To address the second question, we examine temporal differences in metacognitive reflections. Specifically, we investigate how reported strategies may vary across reflection timings. If metacognitive reflections rely solely on physical interactions (physical embodiment hypothesis), similar reports should emerge across real-time and retrospective reflections. In contrast, if retrospective reflection allows for reinterpretation in the absence of direct physical interactions (cognitive embodiment hypothesis), stronger shortcut preferences may emerge under retrospective reflections—particularly in mental jigsaw puzzles, where physical constraints are otherwise salient.

## Materials and methods

### Participants

A total of 82 out of 86 participants completed the main experiment (undergraduate and graduate students from Kyoto University with normal or corrected-to-normal vision). Four participants were excluded: three due to early withdrawal (caused by motion sickness, application failure, or difficulty understanding instructions), and one due to low task accuracy (below 0.70) on the cognitive tasks. Participants were evenly divided into two reflection conditions (*n* = 41 each): the real-time reflection condition [Female/Male = 20/21; Left/Mixed/Right-handed = 7/1/33; Age (years): M = 21.7, SD = 2.8] and the retrospective reflection condition [Female/Male = 21/20; Left/Mixed/Right-handed = 4/1/36; Age (years): M = 21.3, SD = 2.4]. Handedness was assessed using the FLANDERS handedness questionnaire (Nicholls et al., 2013; Okubo et al., 2014). Ethnicity was not collected.

The total sample size is comparable to, or larger than, those used in prior studies examining gender differences in subjective confidence and objective behavioral performance (Cooke-Simpson & Voyer, 2007; Estes & Felker, 2012). Prior to data collection, we conducted a pilot-based chi-square power analysis using the pwr2ppl package (Aberson, 2022). Based on pilot data from six volunteers (laboratory members) (counts: shortcut/detour = 1/5 in FT and 4/2 in MT at 120°; 1/5 in FT and 5/1 in MT at 240°), the analysis suggested that approximately 37 participants per condition would achieve power ≥ 0.80 to detect task differences at 120° and 240°.

## Cognitive task

**Fig. 1:**
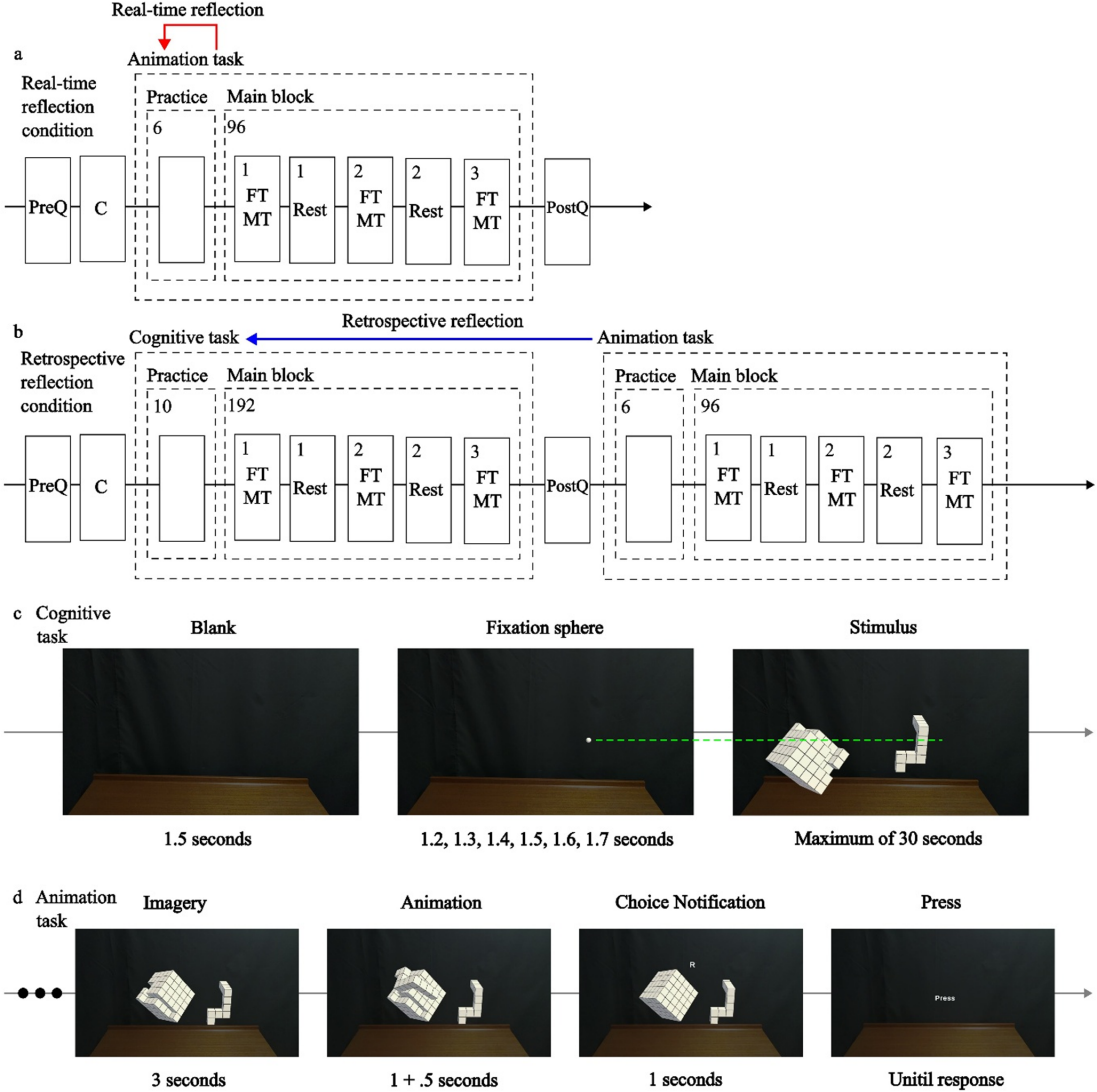
Schematic diagrams of the experiment workflows in **(a)** real time and **(b)** retrospective reflection conditions, **(c)** cognitive tasks, and **(d)** animation tasks. Note. **(a**,** b)** PreQ: handedness questionnaire, C: monitor calibration via eye movements, PostQ: strategy questionnaire, FT: fitting task, MT: matching task. **(c)** The fixation sphere shares the same height as those of the left blank cube and right unit cube of two objects, illustrated by the green dashed line

To replicate the findings previously reported in Yoshioka et al. (2025), the present study employed similar cognitive tasks but adapted them from a computer-screen environment to a mixed-reality setting (Fig. 1c). For feasibility within the available experimental time, the number of stimuli was reduced from 576 to 192. Two tasks—the matching task (MT) and the fitting task (FT)— were used and presented in random order. These tasks required participants to judge whether the right object matched or fit with the left object, respectively. The direction of the required operation (matching or fitting the right object to the left object) was controlled to remain consistent with the directional distinctions highlighted in Yoshioka et al. (2025).

### Animation task

**Fig. 2:**
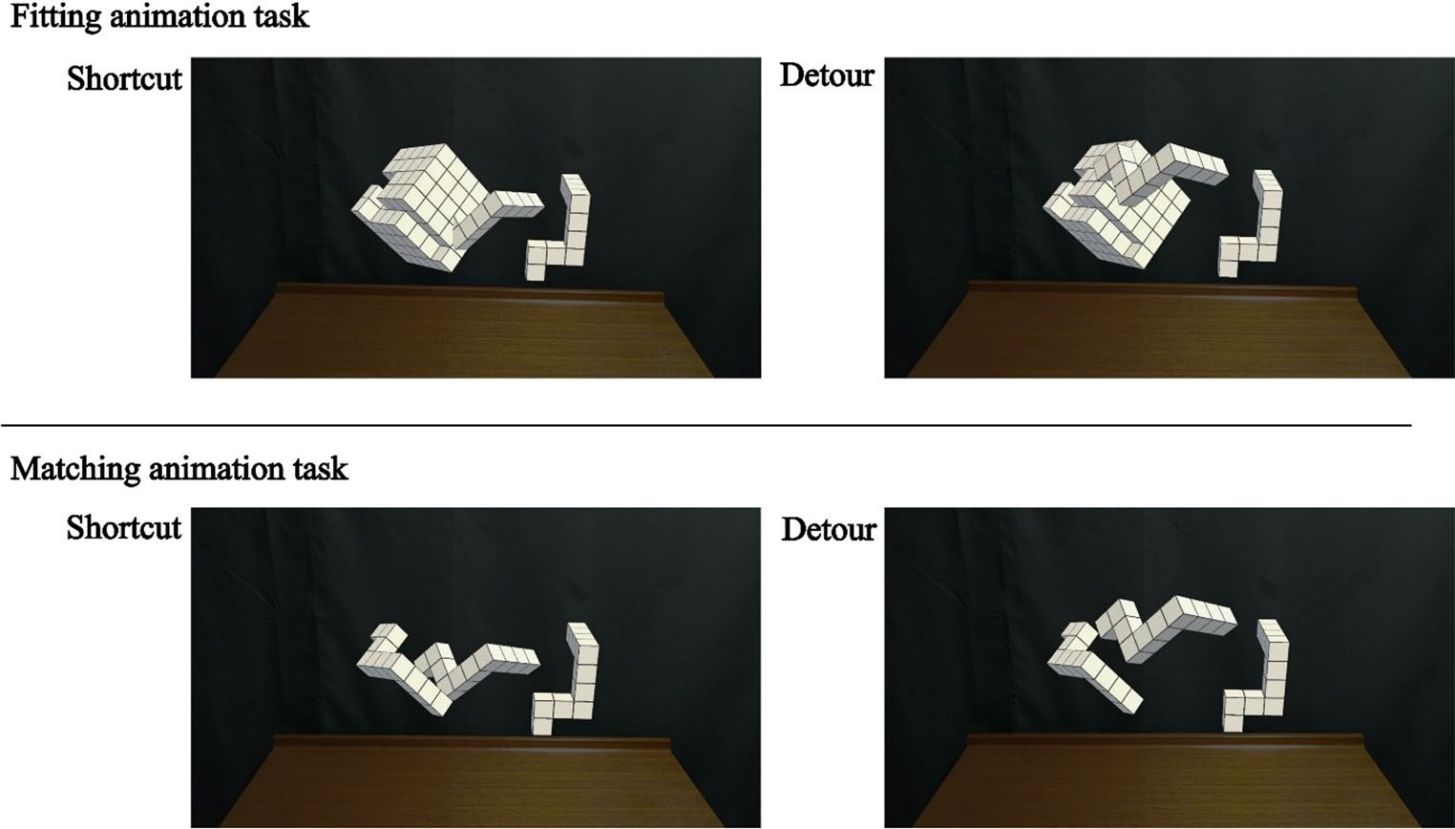
Example frames from the animation task. Note. The shortcut trajectory depicts a direct, straight path toward the target location, whereas the detour trajectory depicts a longer, curved path that deviates from the direct path to avoid an implied spatial constraint. In the fitting animation task, this constraint corresponds to potential physical interference during insertion; in the matching animation task, it is used only as a schematic visualization of an alternative movement strategy rather than a physical obstacle. The objects on the left side are rotated at 240°. These objects are illustrative and differ from the holograms presented in actual experiments via binocular stereoscopic vision. The pieces to be matched or fitted occupy the same space. U-type (larger) objects and T-type (smaller) objects fit together to form a whole cube (Yoshioka et al., 2025)

The animation task used the same congruent object pairs (96 stimuli), presented in random order (Fig. 1d), as prior research—including Gardony et al. (2014)—has shown that congruent and incongruent trials can elicit qualitatively different rotation-related strategies. Unlike the cognitive task, the animation task displayed the dynamic motion of the right object moving toward the left object, involving both rotation and translation, in both the matching and fitting contexts. In each trial, participants viewed two trajectories, also presented in random order: a direct shortcut path and an indirect detour path (Fig. 2).

The animation task was used to assess which translational trajectory participants reflected on under different reflection timings (real-time and retrospective). In the real-time reflection condition (Fig. 1a), participants viewed two objects, imagined matching or fitting the right object to the left object, and then shortly afterward selected the trajectory (shortcut vs. detour) that best represented their strategy for that specific trial. This real-time reflection condition served as the baseline for evaluating whether retrospective reflection differed from real-time reflection judgments.

In the retrospective reflection condition (Fig. 1b), participants first completed the cognitive task, judging whether the right object matched or fit the left object (two-choice responses only; no animation and no subjective strategy reports). After completing all cognitive trials, they were shown the same congruent object pairs again for each trial and asked to imagine performing the same matching/fitting operations they had executed earlier in the cognitive task. They then selected the animation that best represented the strategy they believed they had used in the *previous* cognitive task.

### Stimuli

This study followed the previous methodology (Yoshioka et al., 2025) with bisectional rotation axes and unit cubes but modified other aspects to suit the experimental design. Stimuli were dynamically generated during the experiment, incorporating two types of object pairs (T–T and U–T), four object types, same-mirrored pairs (congruent, incongruent) for the cognitive task, same-object pairs for the animation task, two head types (left, right forward protrusions), and six rotation angles (0° to 300°, in 60° increments). Only the left side was used for object rotation, and object types were reduced by two. To account for grasp regions, specific unit cube positions in both objects were kept at the same height (Fig. 1c). Objects were presented obliquely downward to resemble the two-dimensional images used in previous research (Yoshioka et al., 2025). Object sizes roughly ranged from 11.8° to 14.4° in visual angles.

### Apparatus

The experiment was conducted in an assembled private room equipped with a solid table, a chair, and a desktop light designed to mimic natural daylight while emitting minimal ultraviolet radiation. Participants were seated and wore a HoloLens^®^ 2 headset, a standalone mixed reality device running on Windows^®^ 10. The application used in this experiment was developed using Unity^®^ 2022.3.19f1, in combination with the Mixed Reality Toolkit 3 (MRTK3) and Visual Studio^®^ 2022. Behavioral responses were recorded via a dual-pedal Bluetooth^®^-connected foot switch, placed under the table and paired with the headset. The foot pedal allowed participants to provide responses hands-free during the task.

### Procedure

Figure 1a outlines the procedure for the real-time reflection condition, which included a handedness questionnaire, animation tasks, and a tactics questionnaire (no cognitive tasks). Figure 1b shows the procedure for the retrospective reflection condition, which additionally included cognitive tasks. The total duration of the procedure was approximately 48 min for the real-time reflection condition and 78 min for the retrospective reflection condition. Prior to starting the tasks, participants underwent eye movement calibration and were instructed to interlace their fingers below their stomach to minimize gesture interference (Chu & Kita, 2011).

In the cognitive tasks (Fig. 1c), only participants in the retrospective reflection condition performed object matching and fitting trials. RT and error ratio (ER) were recorded for these cognitive tasks; thus, RT/ER were available only for the retrospective reflection condition. Following a brief practice block (10 trials, with no feedback, in a fixed order, and using stimuli not included in the main blocks), the main block commenced (192 trials). They indicated their responses using a foot pedal: pressing the right pedal if the right block matched or fit with the left block, and pressing the left pedal if it did not. They were instructed to solve the tasks by matching or fitting the right block with the left block, from right to left. This consistent directionality was employed to control for previously reported directional biases in mental rotation and mental jigsaw puzzle tasks (Yoshioka et al., 2025). Participants were instructed to respond as quickly and accurately as possible and to maintain fixation on a sphere between trials.

The animation tasks (Fig. 1d) followed a similar structure to the cognitive tasks but presented animated visualizations of object movement, using only congruent object pairs. Following a brief practice block (six trials, with no feedback, in a fixed order, and using stimuli not included in the main blocks), the main block commenced (96 trials). On each trial, participants completed two imagery–animation sequences. For each sequence, after two objects were presented, participants imagined matching or fitting the right block to the left block, viewed one animated trajectory of the right-side object (T-type block) moving toward the left-side object (U-type or T-type, depending on condition), and received a letter label (L for the first trajectory and R for the second). The assignment of shortcut versus detour to the first (L) or second (R) trajectory was randomized across trials. Finally, participants indicated which L- or R-labeled animation best represented their strategy using a left (L) or right (R) foot-pedal response. In the real-time reflection condition, this imagery was performed for the current trial; in the retrospective reflection condition, participants imagined performing the same matching/fitting operations they had executed earlier during the cognitive task. These animations were designed to reflect plausible mental imagery strategies. In both reflection conditions, participants were explicitly instructed to answer honestly.

In the straight trajectory, a specific reference point on the moving object—typically the ‘neck’ part of the T-type piece that participants were likely to grasp—moved linearly such that this point aligned vertically with the corresponding point on the target object (as shown by the green dashed line in Fig. 2c). In contrast, in the curved trajectory, this same point followed a smooth arc that rose above the green dashed line, tracing a detour-like motion path that avoided an implied spatial obstacle. The selection of this reference point was in line with preliminary hand-tracking data from an ongoing study, in which 7 out of 8 volunteers (laboratory members) were observed to consistently pinch this part of the object.

Each animation consisted of frame-by-frame movement rendered to appear as a continuous, smooth animation. The duration of the animated movement was fixed at 1 s, and the maximum height of the curved trajectory ranged approximately between 48 and 77 mm, depending on the rotation angle. Importantly, the same movement trajectories (straight and curved) were used for both fitting and matching animation tasks, allowing direct comparisons.

The strategy questionnaire employed a two-alternative forced-choice format. Participants in both the real-time and retrospective reflection conditions responded to items concerning strategy use and perceived task difficulty on a tablet computer. They were explicitly instructed to answer honestly. All items and response options were randomized to minimize potential bias. The questionnaire was exploratory in nature, intended to identify potential trends in participants’ self-reports, and is provided on the Open Science Framework (OSF) platform as supplementary information.

### Design

This study employed a mixed factorial design, incorporating both within- and between-participants variables. The reflection condition (Reflection: real-time vs. retrospective) was manipulated between participants, while angle (Angle: 0°, 60°, 120°, 180°, 240°, 300°) and task type (Task: FT, MT) were manipulated within participants. The primary response measure was the self-reported trajectory proportion from the animation tasks, whereas the behavioral measures consisted of RT and ER from the cognitive tasks.

### Data curation

RTs were measured at the third step in Fig. 1c, and were curated using a similar procedure to Yoshioka et al. (2025). The procedure removed the following: durations over 30 s, incorrect responses, responses under 200 msec, approximately the minimum simple RT threshold as reported by Woods et al. (2015), and outliers identified using the median absolute deviation (MAD) method with a threshold of 3 (Leys et al., 2013). The 30-second cutoff corresponded to the trial timeout and was chosen as a conservative upper bound to accommodate slow responses at the largest angular disparity (i.e., 180°) while excluding trials likely reflecting non-task-related delays (approximately 0.064% of all trials). After curation, approximately 82.4% of the congruent trials were retained for calculating mean correct RT. Analyzing only the congruent condition is consistent with the classical mental rotation literature, beginning with Shepard and Metzler (1971) and previous research (Yoshioka et al., 2025). Prior work has also suggested that congruent and incongruent trials may invoke qualitatively different strategies (Gardony et al., 2014). ER was calculated as the ratio of error counts to total counts, with missing data omitted.

The self-reported translational trajectory proportion was calculated as the proportion of detour animations selected out of the total choices (eight trials per condition), with a value of 0 indicating exclusive shortcut selection and 1 indicating exclusive detour selection. The self-reported trajectory proportions and ER were transformed into aligned rank values, following the aligned rank transform (ART) procedure (Wobbrock et al., 2011).

### Data analysis

Statistical analyses involved repeated measures analysis of variance (RM-ANOVA) with Greenhouse–Geisser corrections when sphericity was violated. Effect sizes for ANOVA effects are reported as partial eta squared ($\:{{\upeta\:}}_{P}^{2}$), and generalized eta squared ($\:{{\upeta\:}}_{G}^{2}$) is additionally reported where possible to facilitate comparisons across designs (Olejnik & Algina, 2003). We also conducted ART-ANOVA (Wobbrock et al., 2011). Initially, ART ANOVA were planned to analyze the main effects and interactions including interactions between Reflection and Angle for the self-reported trajectory proportions. However, preliminary analyses indicated concerns about the validity of using ART ANOVA for this dataset. Specifically, the *F*-values not of interest in the aligned responses were not consistently near zero (Kay et al., 2021; Wobbrock et al., 2011), suggesting incomplete alignment of the data. Given these concerns, a nonparametric alternative, the Wilcoxon rank-sum test, was employed to compare reflection conditions.

Within-participant factors were analyzed using RM-ART-ANOVA with effect sizes (Kay, 2021; Wobbrock et al., 2011) and ART-contrasts (ART-C) contrast test (Elkin et al., 2021). All tests were two-tailed, with priority-based and Bonferroni–Holm *p*-value corrections applied where appropriate. All analyses and visualizations were conducted using R software (version 4.4.3) (R Core Team, 2025). The following packages supported the analysis workflow: rstatix (version 0.7.2) (Kassambara 2023a), ARTool (version 0.11.1) (Kay et al., 2021; Wobbrock et al., 2011), ggpubr (version 0.6.0) (Kassambara 2023b), and gridExtra (version 2.3) (Auguie, 2017).

## Results

### Angular pair differences in metacognitive reflections

Here, we focus on mirror-symmetric angle pairs (i.e., 60° vs. 300°, 120° and 240°), which represent spatially equivalent rotations in opposite directions. Any systematic difference in metacognitive reports between such pairs indicates an asymmetry in how participants perceive or evaluate their own strategies. In the retrospective reflection condition, the two-way RM ART-ANOVA for Angle showed a significant interaction effect between Angle and Task [*F*(5, 440) = 3.943, *p* =.002, $\:{{\upeta\:}}_{P}^{2}$ = 0.043]. The post hoc ART-C showed no significant pair differences between 60° and 300° [*t*(440) = 0.120, *p* =.905], or between 120° and 240°, in MT [*t*(440) = 1.935, *p* =.107]. However, in FT, aligned ranks were significantly higher at 300° than 60° [*t*(440) = 2.461, *p* =.028], and at 240° than 120° [*t*(440) = 2.401, *p* =.017].

### Task differences in metacognitive reflections

To examine whether task context (FT vs. MT) influenced metacognitive reflections, we conducted pairwise comparisons at each angle using post hoc ART-C. These comparisons tested whether the proportion of detour responses (reflected in aligned ranks) differed significantly between FT and MT for each angular condition. In the retrospective reflection condition, FT showed significantly higher aligned ranks than MT at all six angles [0°, *t*(440) = 6.620, *p* <.001; 60°, *t*(440) = 4.335, *p* <.001; 120°, *t*(440) = 3.227, *p* =.003; 180°, *t*(440) = 3.746, *p* =.001; 240°, *t*(440) = 7.564, *p* <.001; 300°, *t*(440) = 6.677, *p* <.001].

### Temporal differences in metacognitive reflections

To assess whether reported strategy use differed between timing of reflections (real-time vs. retrospective), we compared self-reported translational trajectory proportions between the two reflection conditions at each angle using the Wilcoxon rank-sum tests.

**Fig. 3:**
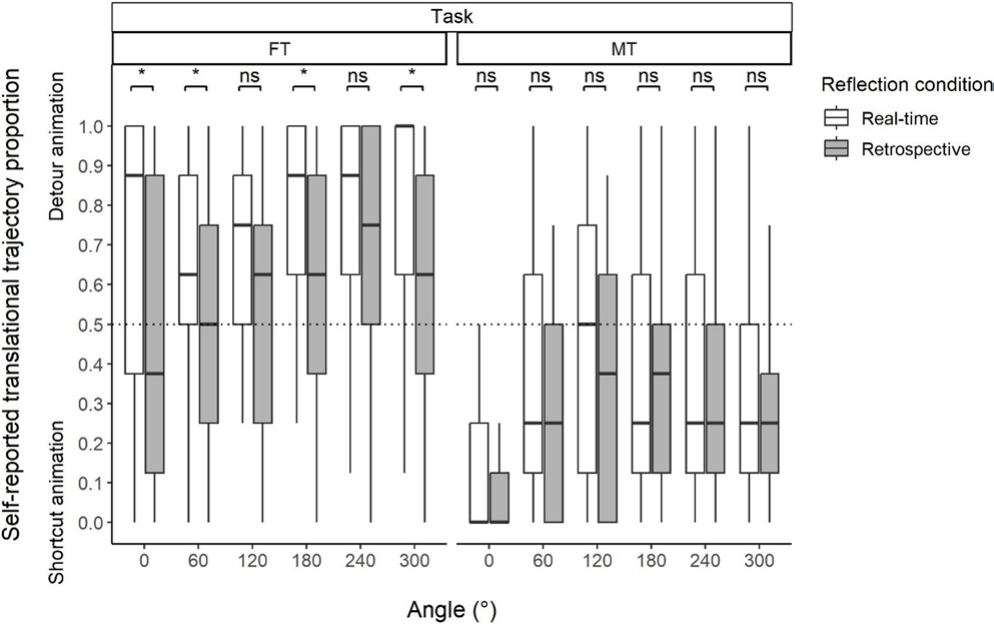
Reflection condition differences at the task level for self-reported translational trajectory proportions across angles. Note. Graphs are described as Median (IQR). FT: fitting task, MT: matching task. ns: p ≥.05, ^*^p <.05, ^**^p <.01, ^***^p <.001

Figure 3 illustrates reflection condition differences at the task level for self-reported translational trajectory proportions across angles. As shown in Fig. 3, the real-time reflection condition reported significantly higher detour proportions than the retrospective reflection condition in FT at angles of 0°, 60°, 180°, and 300° [0°, W = 1124.5, *p* =.031; 60°, W = 1124.5, *p* =.024; 180°, W = 1161.0, *p* =.015; 300°, W = 1128.5, *p* =.030]. Differences at other angles were not significant [120°, W = 1064.0, *p* =.072; 240°, W = 1003.5, *p* =.123]. In contrast, no significant differences were found between reflection conditions in MT at any angle [0°, W = 894.5, *p* =.575; 60°, W = 1027.0, *p* =.484; 120°, W = 969.5, *p* >.999; 180°, W = 774.5, *p* >.999; 240°, W = 925.0, *p* >.999; 300°, W = 923.5, *p* >.999]. Table 1 provides descriptive statistics.

**Table 1:** Descriptive statistics of self-reported translational trajectory proportions

Task	FT		MT	
Angle/Reflection	Real-time	Retrospective	Real-time	Retrospective
0°	0.875 (0.625)	0.375 (0.750)	0 (0.250)	0 (0.125)
60°	0.625 (0.375)	0.500 (0.500)	0.250 (0.500)	0.250 (0.500)
120°	0.750 (0.375)	0.625 (0.500)	0.500 (0.625)	0.375 (0.625)
180°	0.875 (0.375)	0.625 (0.500)	0.250 (0.500)	0.375 (0.375)
240°	0.875 (0.375)	0.750 (0.500)	0.250 (0.500)	0.250 (0.375)
300°	1 (0.375)	0.625 (0.500)	0.250 (0.375)	0.250 (0.250)

Note. Values are described as Median (IQR). Values range from 0 (exclusive shortcut selection) to 1 (exclusive detour selection). *FT* fitting task, *MT* matching task, *Reflection* reflection condition

### Behavioral outcomes (Replication)

#### Correct RT

**Fig. 4:**
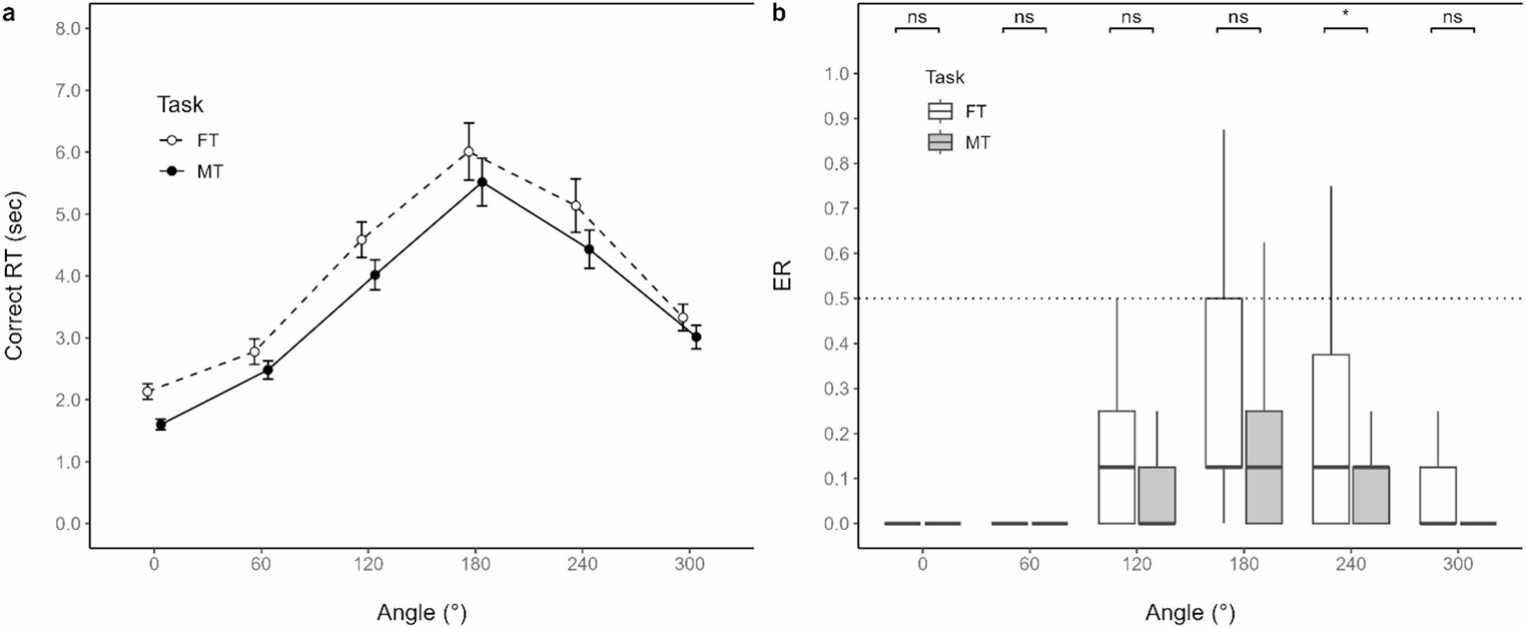
Task differences across angles for **(a)** correct RT and **(b)** ER. Note. **(b)** The graph is described as Median (IQR). ER: error ratio, FT: fitting task, MT: matching task. ns: p ≥.05, ^*^p <.05, ^**^p <.01, ^***^p <.001

Figure 4a illustrates task differences and angle-pair differences for correct RT in the retrospective reflection condition, respectively. The two-way RM-ANOVA showed no significant interaction between Angle and Task [*F*(2.40, 96.05) = 0.456, *p* =.671, $\:{{\upeta\:}}_{G}^{2}$ = 0.002, $\:{{\upeta\:}}_{P}^{2}$ = 0.011], but significant main effects of Angle [*F*(2.22, 88.84) = 80.325, *p* <.001, $\:{{\upeta\:}}_{G}^{2}$ = 0.357, $\:{{\upeta\:}}_{P}^{2}$ = 0.668] and of Task [*F*(1, 40) = 13.946, *p* =.001, $\:{{\upeta\:}}_{G}^{2}$ = 0.018, $\:{{\upeta\:}}_{P}^{2}$ = 0.259]. The post hoc paired Student t-test, after collapsing tasks, showed significantly higher RTs at 300° than 60° [*t*(40) = 4.939, *p* <.001, *d*_z_ = 0.771], and at 240° than at 120° [*t*(40) = 3.031, *p* =.004, *d*_z_ = 0.473]. Table 2 provides descriptive statistics. FT generally took more time overall than MT, and behavioral asymmetry was observed.

**Table 2:** Descriptive statistics of correct RT and ER

Angle/Task	Correct RT		ER	
	FT	MT	FT	MT
0°	2,133 (798)	1,601 (546)	0 (0)	0 (0)
60°	2,777 (1,326)	2,483 (946)	0 (0)	0 (0)
120°	4,586 (1,837)	4,018 (1,538)	0.125 (0.250)	0 (0.125)
180°	6,009 (2,950)	5,516 (2,484)	0.125 (0.375)	0.125 (0.250)
240°	5,136 (2,751)	4,431 (1,988)	0.125 (0.375)	0.125 (0.125)
300°	3,329 (1,372)	3,015 (1,213)	0 (0.125)	0 (0)

Note. Values are described as Mean (SD) in correct RT and Median (IQR) in ER. The units are in msec in correct RT. *RT* reaction time, *ER* error ratio, *FT* fitting task, *MT* matching task

A trend analysis, after collapsing angles from 0°–360° to 0°–180° ranges following previous procedures (Yoshioka et al., 2025), showed significant linear relationships between angular disparity and correct RT for both FT [*F*(1, 120) = 186.98, *p* <.001, $\:{{\upeta\:}}_{\:}^{2}$ = 0.373] and MT [*F*(1, 120) = 262.95, *p* <.001, $\:{{\upeta\:}}_{\:}^{2}$ = 0.468]. While FT demonstrated significant linearity, the angular disparity explained approximately 9.6% less variability in correct RT compared with MT.

#### ER

Figure 4b illustrates task differences and angle-pair differences for ER in the retrospective reflection condition, respectively. The two-way RM-ART-ANOVA showed a significant interaction between Angle and Task [*F*(5, 440) = 6.064, *p* <.001, $\:{{\upeta\:}}_{P}^{2}$ = 0.064]. Post hoc ART-C were conducted to explore this interaction. For task differences at each angle, aligned ranks were significantly higher in FT than in MT only at 240° [*t*(440) = 2.863, *p* =.026]. Differences at other angles were not significant [0°, *t*(440) = 0.201, *p* =.841; 60°, *t*(440) = 0.985, *p* =.650; 120°, *t*(440) = 1.535, *p* =.376; 180°, *t*(440) = 2.100, *p* =.145; 300°, *t*(440) = 2.156, *p* =.158]. For angle pair comparisons, aligned ranks were significantly higher at 300° than 60° [*t*(440) = 2.786, *p* =.011], and at 240° than 120° in FT [*t*(440) = 2.273, *p* =.024], whereas no such differences were found in MT between 300° and 60° [*t*(440) = 1.615, *p* =.214], nor between 240° and 120° [*t*(440) = 0.945, *p* =.345]. Table 2 provides descriptive statistics. Task differences were less pronounced overall, and behavioral asymmetry was observed only in FT.

## Discussion

This study primarily aimed to examine two core research questions concerning metacognitive reflection: **(1)** which type of translational trajectory participants retrospectively report (shortcut vs. detour; minimum-distance vs. physical-simulation hypotheses), and **(2)** whether these reflections are malleable across interpretive conditions (real-time vs. retrospective reflections; cognitive vs. physical embodiment hypotheses). In addition, to assess replicability, this study examined RT patterns to determine whether previously reported behavioral asymmetries could be replicated. Finally, this study explored the relationship between RT patterns and subjective strategy reports to evaluate their alignment in mental translation.

The first research question concerned which translational trajectory participants retrospectively reflect on during FT and MT (shortcut vs. detour; minimum-distance vs. physical-simulation hypotheses). Analyses of angular-pair differences in FT revealed that participants in the retrospective reflection condition showed a bias toward selecting detour animations when the orientation of the U-type object suggested greater obstruction—particularly on the thick-wall side (e.g., 300°) compared to the thin-wall side (e.g., 60°). In contrast, no similar pattern emerged in the MT involving T-type object pairs, in which configurations lacked clear physical obstacles. These results suggest that participants’ retrospective trajectory reflections were influenced by physical constraints inherent in object pairs—specifically, the functional distinction between a piece-to-piece (MT) versus a piece-to-assembly (FT) context. The asymmetric pattern of trajectory reflections around 180° observed in FT aligns with the physical simulation hypothesis.

Task differences in retrospective reflections were also examined. Across all angles, participants showed a bias toward selecting detour trajectories significantly more often in FT than in MT. The strongest effects occurred at angles with spatial configurations of thicker barriers—most notably 240°, where the median difference in detour preference between FT and MT reached 0.500. In contrast, the difference was smaller at angles such as 120° (0.250). Although descriptive, these findings indicate that participants relied more heavily on spatially grounded reasoning in FT compared to MT, potentially due to the functional roles implied by the object configurations. In particular, the fitting context may have prompted participants to reflect on physical constraints—such as how a piece fits into an assembly (i.e., the piece-to-assembly relationship)—which extend beyond simple geometric alignment. The heightened detour preference in FT relative to MT indicates greater reliance on the physical simulation hypothesis in FT compared to MT.

Earlier studies employing puzzle-like stimuli (Frick et al., 2013; Frick & Pichelmann, 2023; Mutlu et al., 2020) often conflated the cognitive processes of “matching” and “fitting,” potentially obscuring important functional distinctions. These studies used fully framed and perceptually complete stimuli, which may have encouraged perceptual matching rather than the type of spatial reasoning required in real-world puzzle fitting. In contrast, the present mixed-reality study employed incomplete, partially framed shapes, offering a more ecological design and, to our knowledge, demonstrating for the first time how mental jigsaw puzzles can elicit distinct metacognitive patterns compared to classical mental rotation tasks.

At the same time, it is important to interpret these differences not only in relative terms (FT compared to MT) but also in absolute terms within FT itself. Within FT in the retrospective condition, detour trajectories were not uniformly preferred—at angles such as 60°, shortcut and detour selections occurred at comparable rates. This pattern is compatible with both the physical simulation hypothesis and the minimum-distance hypothesis, yet it contrasts with everyday physical behavior, in which fitting an object into a constrained space often requires a detour-like motion. Such deviations indicate that trajectory reflections may not solely reflect real-world physical-constraint reasoning, but may instead be shaped by task-induced cognitive reinterpretations. This possibility is examined next by comparing real-time and retrospective reflection contexts.

The second research question concerned whether these reflections are malleable through cognitive reinterpretations (real-time vs. retrospective reflections; cognitive vs. physical embodiment hypotheses). Analyses of temporal differences in metacognitive reflections revealed no reliable real-time–retrospective differences in MT. In contrast, in FT, the retrospective reflection condition showed lower detour proportions (i.e., a shift toward shortcut selections) relative to the real-time reflection condition at most angles (e.g., 60°)—even under configurations suggesting spatial constraints (0°, 180°, 300°; Fig. 3), where detour strategies would seem more appropriate in physical puzzle solving. The real-time–retrospective contrast in FT was especially marked at angles with implied obstacles (0° and 300°), with median differences in detour preference of 0.500 and 0.375, respectively; by comparison, only a minor difference (0.125) was observed at 60°. While the data remain descriptive, the observed shift suggests that engagement in cognitively demanding tasks under speed–accuracy emphasis may lead participants to reinterpret their metacognitive reflections, potentially emphasizing efficiency and directness over more indirect, obstacle-sensitive planning. This complicates any strict dichotomy in trajectory reflections between matching (geometric efficiency; associated with MT) and fitting (physical simulation; associated with FT). The convergent tendency of reflection reports across FT and MT—consistent with preliminary implications from a pilot study (Yoshioka, 2023)—suggests that not only physical interactions (e.g., real puzzle solving) but also purely cognitive interactions (i.e., fast-paced visual tasks without physical interaction) may embody cognition, shaping how strategies are retrospectively reflected on, which is consistent with the cognitive embodiment hypothesis.

At first glance, the shortcut-oriented preference observed in retrospective reports may seem inconsistent with a classical affordance account (Gibson, 1979), which posits that objects invite potential motor actions such as detouring around obstacles. However, it is important to clarify that traditional affordance theory primarily concerns perceptual-motor interactions rather than metacognitive processes. Thus, the current findings neither directly support nor contradict affordance theory in its original formulation. Rather, as noted earlier, embodied cognition—particularly through cognitive interactions—may have reinterpreted participants’ metacognitive reflections. Specifically, the converging tendency of metacognitive responses in FT, shifting from detour trajectories during real-time reflection toward more shortcut-oriented retrospective reports, may reflect an assimilation of task interpretations from physically grounded reasoning toward a more abstract, efficiency-oriented framing.

In contrast to FT, metacognitive reports in MT showed little evidence of interpretive malleability: participants largely maintained a shortcut-oriented preference. Notably, closer inspection of Fig. 3 suggests that participants exhibited a roughly increasing tendency to select detour reflections as angular disparity increased—echoing the classical assumption that RTs increase monotonically across angles (i.e., a symmetric function around 180°). It remains unclear why participants would prefer such reflections even when the shortest translational path was clearly depicted in the animation. One possibility is consistent with the “box” interpretation proposed by Yoshioka et al. (2025): participants may have mentally reframed the T-type object as if it were enclosed within an implicit U-shaped boundary, thereby introducing a perceived “entry point” and encouraging detour-like reflections at larger angular disparities, even when multiple entry points are available.

Overall, these findings offer meaningful insight into participants’ metacognitive interpretations of their spatial strategies. At the same time, to better interpret these reflections, it is necessary to establish whether the underlying behavioral patterns are replicated in the present setting. Accordingly, before turning to the relationship between behavior and reflection, we first examine whether the previously reported RT asymmetries can be replicated in the present mixed-reality environment.

With respect to replication, participants in the retrospective reflection condition generally exhibited longer RTs in FT than MT. Furthermore, RTs differed for angular pairs with equivalent angular disparity (e.g., + 60° and − 60°), producing a behavioral asymmetry around 180°; RTs were longer at 300° (− 60°) than at 60° (+ 60°) in both FT and MT. These results replicated the previous findings by Yoshioka et al. (2025). While this asymmetry is readily interpretable in mental jigsaw puzzles as reflecting detour-like strategies under implied physical constraints, its presence in classical mental rotation tasks remains theoretically difficult to explain. One possible account is that translational components (i.e., detours) contribute to RTs even in mental rotation tasks, extending the classical assumption that rotational distance (i.e., angular disparity) determines RTs to include translational distance as well. For example, participants might mentally represent object boundaries in a way that effectively transforms T–T object pairs into U–T or T–U configurations—a so-called “box” interpretation (Yoshioka et al., 2025)—thereby making MT functionally similar to FT. However, such “virtual boxes” can be construed in multiple ways depending on how potential entry points are imagined, and this account also lacks a clear rationale in the absence of actual physical constraints. It therefore remains difficult to conclude that participants consistently respected physical constraints in MT.

An alternative interpretation is that RT asymmetries reflect decision-level processes—such as conceptual coherence between imagined rotation and translation—rather than the computational cost of spatial manipulations itself (Yoshioka et al., 2025). This view is supported by the observation that the RT increase from 0° to 180° were substantial [approximately 3.9 s in the present study, compared with roughly 2.3 s in the previous study (Yoshioka et al., 2025)], far exceeding what would plausibly be required to mentally—or even physically—rotate a three-dimensional object. Moreover, prior work using sequentially cued tasks suggests that translation is executed rapidly at the cognitive level (Cave et al., 1994), further implying that RTs may be dominated by processes other than the mental manipulations per se.

A further complication concerns the pronounced “dip” in RTs at 60°. Under extended distance-based accounts (i.e., costs that scale with rotational and translational distances), RTs should increase linearly from 0° to 180° along the shortest path, or lie above this function if a longer detour path is taken. Although Yoshioka et al. (2025) suggested that elevated RTs at 0° might reflect object-recognition demands—thereby creating an apparent dip at 60°—this explanation is insufficient. Specifically, their data showed a dip at 60° on the left-rotation side but a corresponding bump at 60° on the right-rotation side, indicating that the phenomenon cannot be attributed solely to processes occurring at 0°. Such dip behavior is more readily accommodated by conceptual-coherence accounts, in which performance is facilitated when the imagined laterality codes of translational and rotational directions align (e.g., a leftward translation paired with a counterclockwise rotation, yielding a left–left alignment) (Yoshioka et al., 2025).

Taken together, although the present replication confirms the robustness of asymmetric RT patterns, it also reinforces a fundamental limitation of RT-based analyses: behavioral measures alone are insufficient to determine whether participants actually engage in shortcut or detour-like mental trajectories. The same asymmetric RT pattern may plausibly arise from multiple underlying processes, including distance-based manipulation costs and decision-level coherence effects, the latter of which can also account for the observed dip behavior. This ambiguity motivates further investigation through the incorporation of metacognitive reflection. In the seminal work by Shepard and Metzler (1971), the alignment between RT patterns (monotonic linear increases across angular disparity) and participants’ self-reports of mentally rotating objects was taken as evidence for mental rotation processes. By extension, examining the alignment between metacognitive reflection on translational trajectories and RT patterns may provide insight into mental translation processes.

With respect to the relationship between behavioral performance and strategy reports, a clear discrepancy emerged. The behavioral measure (RT) showed a main effect of Task, with both tasks exhibiting asymmetric patterns. In contrast, the subjective measure (self-reported translational trajectory proportion) revealed an interaction effect between Task and Angle, with an asymmetric pattern in FT but a symmetric pattern in MT. This dissociation departs from the implication drawn from Shepard and Metzler (1971)—namely, that subjective reports of mental rotation should correspond closely to objective performance. Importantly, this assumed correspondence itself presupposes the classical behavioral expectation that equal angular disparities yield equivalent RTs (i.e., a symmetric RT–angle function). The present divergence therefore challenges the presumed alignment between introspective and behavioral measures. Several interpretations remain possible: **(1)** subjective reports of translational trajectories may be more reliable than RTs; **(2)** RTs may be more reliable than subjective trajectory reports; or **(3)** both measures may be limited in different ways.

First, as discussed earlier, RTs may not directly capture shortcut or detour trajectories because the underlying mental rotation or translation processes may be cognitively rapid, or because the observed RT patterns may instead reflect an alternative mechanism (e.g., conceptual coherence). In this context, metacognitive reflections may offer more insight into internal strategy use. Indeed, prior work reported that confidence can capture performance in mental rotation tests, with moderate to strong confidence–performance correlations (Cooke-Simpson & Voyer, 2007; Estes & Felker, 2012). Unlike RTs—which aggregate multiple processes, including decision-making and motor execution—participants’ strategy reports focus directly on the nature of the manipulation itself (e.g., whether they imagined detouring around implied obstacles). The stronger interactions between task and angle observed in metacognitive reports may therefore better reflect participants’ conceptualization of spatial strategy, being more intuitive as detour reflections in obstacle-rich contexts and as shortcut reflections in obstacle-scarce contexts. From this view, metacognitive reports may more selectively highlight functional differences between FT and MT.

Second, however, an alternative interpretation must also be considered: that RTs may more faithfully reflect actual cognitive strategy use, whereas metacognitive judgments may be vulnerable to distortions such as overconfidence (Fischhoff et al., 1977). From this perspective, participants may have engaged in similar strategies across FT and MT. For example, as discussed earlier, Yoshioka et al. (2025) proposed that MT may elicit detour-like trajectories similar to those in FT. In real physical environments, perfect spatial alignment between two objects is impossible, as physical boundaries constrain object shapes. Participants might, therefore, mentally simulate a detoured path in MT as well—such as by imagining a virtual “tight box” surrounding the object, effectively transforming the T-shaped object into a U-shaped one, resulting in a curvier trajectory similar to that expected in FT (Yoshioka et al., 2025). If such strategies were adopted in both tasks, this would explain the main effect of Task observed in RTs without interactions between task and angle. The differences in RTs might then arise not from distinct strategies, but from other factors such as object recognition.

In contrast, during metacognitive reflection, participants may lack direct access to the strategies they actually employed. This possibility is consistent with nondeclarative procedural memory, in which skilled performance is executed without conscious recollection (Roediger, 1990; Squire, 2004). By extension, the mental operations used during spatial reasoning may likewise become proceduralized, rendering them opaque to introspection while they unfold. As a result, participants may not accurately retrieve how they carried out the underlying mental manipulation and may instead rely on surface-level task features or assumptions about what the task “should” involve. Indeed, accessing cognitive processes through verbalization may influence performance (Ericsson & Simon, 1993). Similarly, attempts to introspect on cognitive processes may alter or reconstruct those processes retrospectively. In the case of FT, the presence of visually salient, obstacle-like structures may lead participants to infer that a detour strategy was used—even if no such strategy was consciously enacted. Such reliance on external cues or normative expectations may distort introspective access and contribute to divergences between RT-based performance measures and subjective reports of strategy use.

Third, it remains possible that neither RTs nor retrospective reflections accurately capture the underlying trajectory. Although both measures appeared to support the use of detour trajectories in FT, these findings may reflect misinterpretations rather than genuine mental trajectories. This is because FT requires only determining whether the right object can fit with the left object—that is, whether the start and end spatial coordinates are compatible. The task itself does not require mentally simulating a longer, detoured path via the mental representation of physical obstacles. Consequently, both RTs and reflective reports may have inferred detours that were not actually part of the underlying cognitive process, indicating that these measures alone may not fully capture the true nature of the trajectory. Further investigation is therefore needed.

Taken together, although the present study revealed a clear divergence between objective performance and subjective reports, the present study cannot determine which interpretation more captures translational trajectories. Nonetheless, the findings challenge the longstanding intuitive assumption—central to Shepard and Metzler (1971)—that objective performance and introspective reports necessarily align as converging evidence for the presence of analogue-like, continuous mental rotation.

### Limitations

While this study offers valuable insights into how perceived physical constraints influence metacognitive reflections on cognitive strategies during mental jigsaw puzzle tasks, several limitations should be acknowledged. First, sample-size planning relied on a small pilot study (*n* = 6) and chi-square–based power calculations using binary questionnaire responses. Accordingly, the resulting estimate should be interpreted cautiously and may not fully capture angle-specific or reflection-related effects observed in the main analyses. In the present study, strategy reports were measured as proportions derived from eight trials per condition, providing finer resolution than the pilot. Future research would benefit from more rigorous power analyses based on a larger number of pilot participants and more continuous outcome measures to improve the robustness and generalizability of the findings.

Second, strategy reports were constrained to a two-alternative forced-choice between predefined whole-object trajectories. Although whole-object (holistic) treatments have been used in prior work (Gardony et al., 2014; Wohlschläger & Wohlschläger, 1998), this format may not capture piecemeal or locally defined strategies suggested by prior eye-movement and behavioral work (Just & Carpenter, 1976; Pylyshyn, 1979; Xue et al., 2017). Nevertheless, piecemeal approaches may still involve translational movement of object components, providing some basis for interpreting the shortcut and detour options. We attempted to mitigate this constraint by instructing participants to choose the animation that *best* represented their strategy rather than one that perfectly matched it.

Third, task duration differed between the real-time and retrospective reflection conditions (approximately 48 vs. 78 min, including administrative procedures such as informed-consent instructions). This difference may have introduced condition-specific effects of stimulus exposure/familiarity, fatigue, and practice-related learning, particularly in the retrospective reflection condition, which included the cognitive tasks prior to the reflection judgments. Future studies may investigate the factors driving the retrospective (vs. real-time) shift toward more shortcut-oriented selections in FT.

### Future directions

Several promising avenues for future research emerge from the findings and limitations discussed in this study. First, future experiments may directly investigate the role of conceptual coherence—particularly how the directions of mental translation and rotation interact. Systematic manipulation of these directions would clarify their combined influence on cognitive strategies and help resolve whether the observed behavioral asymmetries result primarily from conceptual coherence or from other cognitive factors.

If this coherence relationship is confirmed, it may offer a new avenue for investigating trajectory representation. For example, previous research has employed rotational motion aftereffect paradigms to show that directional coherence between mental rotation and the induced aftereffect can facilitate, whereas directional incoherence can delay, RTs (Corballis & McLaren, 1982; Heil et al., 1997). Building on this, future studies could manipulate the direction of translational motion—anchoring 0° to linear movement and introducing increasing angular offsets at onset to produce curvier trajectories—to examine their influence via translational motion aftereffects.

Second, exploring the role of embodied cognition through hand-tracking technologies presents a promising research direction. While previous studies have largely overlooked mental translation and its associated trajectories, integrating hand-tracking data may help bridge the gap between mental imagery and physical action. This approach could offer a clearer understanding of how embodied constraints shape cognitive strategies during spatial reasoning tasks. Such an approach is particularly relevant in light of neurological evidence that mental rotation tasks involving hand tools are associated with activation in motor-related cortical regions and with hand dominance (Vingerhoets et al., 2002). Our ongoing research aims to clarify the role of trajectory selection by analyzing patterns of actual hand movements in classical mental rotation tasks and mental jigsaw puzzles.

Third, future research may investigate how metacognitive reflections support learning through mental practice. Mental rehearsal has been shown to enhance subsequent performance (Driskell et al., 1994; Toth et al., 2020). The present findings suggest that the content and timing of metacognitive reflections (i.e., real-time vs. retrospective) influence how individuals conceptualize their strategies. Promoting accurate and timely metacognitive reflections could strengthen the benefits of mental practice in spatial tasks. This line of research may offer new approaches for improving spatial performance by training individuals not only to act but also to reflect effectively.

This possibility may be particularly relevant given prior evidence that beliefs about one’s spatial abilities can narrow—or even eliminate—gender differences in behavioral performance on mental rotation tests (Moè, 2009; Moè & Pazzaglia, 2006). By extension, fostering appropriate reflection on intermediate processes may likewise help reduce such gaps. For example, a secondary analysis indicated a gender-related trend in the real-time reflection condition: female participants tended to reflect more on detour trajectories, a descriptive pattern that was not clearly observed in the retrospective reflection condition (Yoshioka & Saiki, 2025).

## Theoretical contributions and concluding remarks

The present study replicated the asymmetric reaction time patterns in mental rotation tasks and mental jigsaw puzzles reported by Yoshioka et al. (2025), thereby challenging the classical expectation reported by Shepard and Metzler (1971) that equal angular disparities necessarily yield equivalent reaction times in matching objects. Retrospective reports of translational trajectories mirrored this asymmetry in the puzzle task but not in the classical mental rotation task, revealing a dissociation between objective performance and subjective strategy reports. Notably, metacognitive reflections were malleable in the puzzle task, whereas they remained stable in the classical task.

## Data Availability

Raw data and preregistrations are available from OSF platform: https://osf.io/c58av/.
